# Improving adherence to the Mediterranean Diet through a bio-psycho social and sociotype approach

**DOI:** 10.3389/fnut.2023.1232078

**Published:** 2023-09-20

**Authors:** Lorenzo M. Donini, Elliot M. Berry

**Affiliations:** ^1^Department of Experimental Medicine, Food Science and Human Nutrition Research Unit, Sapienza University of Rome, Rome, Italy; ^2^Braun School of Public Health, Hadassah Medical School, Hebrew University, Jerusalem, Israel

**Keywords:** adherence, Mediterranean Diet, sociotype, social environment, institutional context, sustainability

## Introduction

In spite of the very many papers indicating that the Mediterranean Diet (MD) is one of the most healthy and sustainable eating patterns, adherence to it diet is diminishing in most Mediterranean countries.

In the study published by Vilarneau et al. the adherence to the MD over a 50-year period (1961–1965 to 2004–2011) in 169 countries declined in most countries (overall from 2.86 to 2.03 according to the Mediterranean Adequacy Index) in particular the Mediterranean Europe, Southern Mediterranean, and Central Europe countries. These regions have undergone significant cultural, social and political changes, which may have influenced the dietary transition and changes in food habits. Moreover, different studies reported an association between adherence to the MD and socioeconomic factors, with greater wealth being associated with increased adherence to the MD (1–3).

There is therefore a necessity to “revitalize” the MD and to return to a model that is considered a reference for all nutritional guidelines throughout the world (4).

The term *sociotype* describes the reciprocal relationship of an individual with the social environment during life. The sociotype is a theoretical ecological framework to emphasize the bio-psycho-social and environmental factors involved in coping with life stresses (e.g., food insecurity) (5) and patient self-management for chronic illness such as diabesity (6). The sociotype is a framework for helping in coping with different life challenges (7): it has been used for food security (5) and also during the recent COVID shutdown (8). All three domains are involved to different extent in these situations depending on the person and the issues involved. The three domains of the sociotype refer in this paper to: Individual, Relationships/Social environment, and Institutional Context.

**Figure 1:**
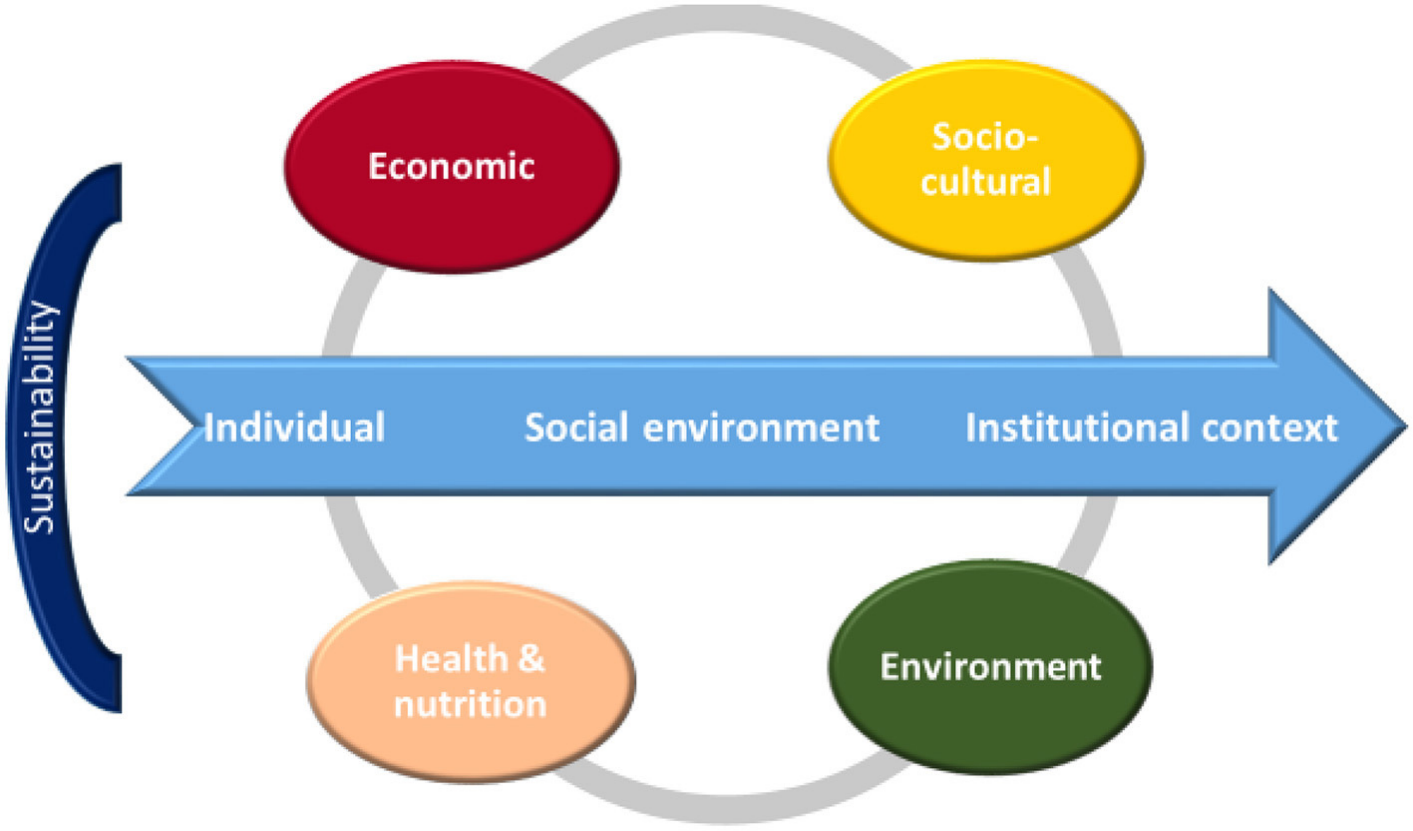
Sociotypic approach to the sustainability of food chain.

The sustainability of the MD has been defined through four dimensions: socio-cultural, economic, environmental, health-nutritional (9). Enhancement of adherence to the MD and its eating pattern should consider (Figure 1):

1) its longitudinal vector—involving the individual called to make healthy and sustainable choices, the family/social relationships and living environment in which these choices must be favored, and the institutional context necessary to promote a such a model for people and the planet and
2) its transversal vector—involving the four domains of sustainability: socio-cultural, economic, environmental, health-nutritional dimensions. Together, these can represent important driving forces to improve adherence to the Mediterranean eating pattern.

The aim of this article is to discuss promoting adherence to the MD by considering the four dimensions of sustainability in an integrating bio-psycho social and sociotypic approach (10).

## Sustainable development—The economic dimension

The economic dimension of sustainability deals with the economic conditions of stakeholders, and on economic systems at local, national, regional and global levels (11). It includes:

1. Economic functioning (generated and distributed economic values).
2. Market presence (wages and social benefits by gender, employment opportunities, number of senior management hired from the local community).
3. Indirect economic impacts (development of infrastructure investments and supported services).
4. Procurement policies (proportion of expenditure on providers at key locales).

It has been shown that MD has signficant economic benefits due to its beneficial influence on the prevention of non-comunicable chronic diseases (12), including obesity, some cancers and degenerative neurological diseases, and thereby reducing health expenditure for individuals and health care systems.

A MD based on local foods promotes the economic valorisation of territories and will keep their traditional products linked to history and culture, thereby boosting business both for local producers and distributors.

In particular, recently, the idea of eating food grown and produced locally has gained much attention since it reduces the environmental and economic impacts of transportation, with a reduction of household spending, and increasing the nutritional value of foods (in particular fruits and vegetables) (13).

The sociotypic approach to enhance the adherence to MD, considering in particular the economic dimension of MD, includes individual commitment, interventions engaging the social environment, and the institutional context as described in Table 1.

**Table 1:** Sociotypic approach to the economic dimension of food chain sustainability.

**Individual**	**Social environment**	**Institutional context**
Sensible food choices considering resources employed for their production (energy, water, land use)	Less expensive foods	Policies to support the consumption of high-priced foods (e.g., olive oil, fish)
Reduce food waste without looking at the aesthetic aspects of food	Ensure sustainable and production patterns	Poverty alleviation, equity, and social justice
Consider correctly the expiration dates of foods and “consume preferably by” indication	Adopt recycling procedures for reducing food waste	Demographic transition: less young, more elderly; Urbanization
Choose cheaper but equally nutritious foods (chicken, milk, eggs, legumes,…)		

## Sustainable development—The socio-cultural dimension

The social dimension of sustainability is based on equal opportunities for all to healthcare and an adequate level of education. Equality and the refusal of any form of discrimination together with the warranty of peace, all contribute to a socially sustainable development. Food security is an essential part of this feature of sustainability.

Social aspects of sustainability are tightly linked to cultural dimensions. A sustained level of culture and (5, 14) traditional knowledge is in fact absolutely necessary to ensure sustainable development, to find the right solutions that align economic considerations with environmental protection and influence politics at a national and international levels (15).

The report “Culture in the implementation of the 2030 Agenda” (https://agenda21culture.net/sites/default/files/culture2030goal_high.pdf) provides key recommendations for all parties involved in the Implementation Decade (2020–2030) of the Sustainable Development Goals (SDGs) considering:

1. including cultural aspects initially in national frameworks for implementing the SDGs;
2. the importance of local culture in the implementation of the SDGs and the critical roles played by the civil society, institutions, and organizations;
3. commitment to developing multi-level partnerships to strengthen the integration of the cultural dimensions of the SDGs.

Territorial diets, such as the MD, are by nature related to specific geographic regions which have over time, assimilated other influences through the transfer of people material and cultural merchandise, including victuals. In keeping with the local cultural, socio-economic, and environmental contexts, territorial diets are linked not only to the biophysical reserves (soils, microclimates, landscape) that define agriculture and economic practices, but also to particular historical contexts, ecologies, and socio-cultural resources including institutions, and traditional knowledge. Examples of such territorial diets include: The Japanese Diet, the Mediterranean Diet, the Traditional Nordic Diet, and the New Nordic Diet (16).

The MD promotes:

- awareness of the local terroir, seasonality, and biodiversity
- traditional and local foods and culinary activities;
- social interactions through conviviality;
- awareness of the entire historical and cultural heritage of the MD—which is s a dietary tradition passed on from generation to generation.

The sociotypic approach to enhance the adherence to MD, considering its the socio-cultural dimension, includes its three domains described in Table 2.

**Table 2:** Sociotypic approach to the socio-cultural dimension of food chain sustainability.

**Individual**	**Social environment**	**Institutional context**
Revive conviviality and culinary activities	Promotion through social media; role models	Public health and nutrition education (in schools, for health care professionals, and policy makers, …)
Awareness of the characteristics of healthy and sustainable diets; improved lifestyle choices	Changing role of working women in the family: time constraints preventing preparation and cooking meals	Fine-tuning and updating dietary guidelines for a healthy and sustainable diet
Adopt more vegetarian eating patterns (more forks than knives)	Role of the family in transmitting food culture and traditional knowledge	Promotion of healthy and sustainable dietary models throughout the catering systems
Choose fresh, home-made foods rather than ultra- processed and fast foods	Promotion of farmers markets	Effective food labeling

## Sustainable development—The health and nutrition dimension

Much research has considered the environmental impacts of various diets, concluding that a plant-based diet, with less animal-sourced foods confers both improved health and environmental benefits—as encouraged by the motto “more forks than knives.”

A healthy diet should optimize health, as defined by a state of complete physical, mental, and social wellbeing and not merely the absence of disease. Healthy diets, such as the MD, have an optimal caloric intake and consist of a wide range of plant-based nutrients, low amounts of animal foods, containing more unsaturated than saturated fats, and with limited amounts of refined grains, highly processed foods, and added sugars (17).

Very many scientific papers (18–21) have demonstrated the health benefits of MD through the prevention of cardiovascular and metabolic diseases, cancer, and depression while slowing the degenerative processes related to aging. In particular Sofi et al. have shown analyzing a global population of nine cohort studies including 514,118 subjects, an increase of two points in the adherence score determined an 8%-protection against a premature death (RR: 0.92, 95% CI 0.90–0.94, *P* < 0.0001) (21, 22).

The sociotypic approach to enhance the adherence to MD, considering in particular the health and nutritional dimension of MD are listed in Table 3.

**Table 3:** Sociotypic approach to the health and nutritional dimension of food chain sustainability.

**Individual**	**Social environment**	**Institutional context**
Empower people to take responsibility for a healthy lifestyle	Decrease screen time [the Am Acad of Pediatrics recommends: (1) no screen time for children under 2 years; (2) 1 h per day for children 2–12 years old; (3) 2 h per day for teens and adults; www.healthychildren.org] and encourage physical activity (https://www.who.int/publications/i/item/9789241599979)	Positive instead of negative nutritional messages
Adopt food choices in line with dietary guidelines	Parental responsibility and education on healthy lifestyle	Creation of playgrounds and spaces suitable for physical activity
Awareness of the early warning signs for eating disorders	Discourage rewarding with food	Encourage breast feeding (up to 90% of exclusive breastfeeding on discharge and 80% at 4 months—https://extranet.who.int/nutrition/gina/en/node/23607)
Limit portion size		High quality health care services

## Sustainable development—The environmental dimension

The food chain has important impacts on the environment through the release of huge amount of carbon dioxide (CO_2_) into the atmosphere, together with water, soil, land use, and energy consumption (23). Moreover, the production of foods has led to deforestation of large parts of the planet while, *pari passu*, biodiversity of plants and animals used for human nutrition has decreased. This has been caused by a progressive homogenisation of eating habits all over the world, and to the lobbying to increase live-stock and the productivity of the agricultural system.

Moreover, in this Anthropocene epoch, the global food system must operate to optimize human wellbeing and food production to ensure, from sustainable food systems, healthy diets for nearly 10 billion people by 2050, while guaranteeing food security, without negatively impacting on the environment. Finally, it is estimated that around a third of the global food production is lost throughout the entire food chain, equally distributed during the production process, along the transportation/conservation/transformation procedures and the consumption sites (including households; https://www.fao.org/in-action/seeking-end-to-loss-and-waste-of-food-along-production-chain/en/).

The MD, as a plant-based dietary model, has demonstrated its environmental benefits that are linked to the reduced use of natural resources (water, soil) and the reduced GHG emissions (13). The use of seasonal products, which is one of the cornerstones of the MD, may contribute to reduce environmental impact of food chain (reduction of greenhouse crops and transport costs from distant countries) and to the preservation/increase of biodiversity (safeguarding small producers, different sowing, and rotation of crops). Frugality (consumption of moderate portions, and of fresh, minimally processed foods) and culinary activities and recipes (in many cases based on the recycling of foods) typical of the MD may contribute to the reduction of food waste.

The sociotypic approach to enhance the adherence to MD, considering the environmental dimension of MD, is described in Table 4.

**Table 4:** Sociotypic approach to the environmental dimension of food chain sustainability.

**Individual**	**Social environment**	**Institutional context**
Consume only seasonal products	Promotion through social media	Promoting resource efficiency
Prefer local food products	Discourage “all-you-can-eat” promotions	Food-banks/food aid
Maintain biodiversity in food choices	Minimizing food loss and waste	Consider sustainability costs for food price policies
Consume only what is needed		

## Conclusions

The bio-psycho-social and sociotypic approach to the multi-faceted nature of the MD can enable nutritionists and policy makers to focus on the different domains—Individual, Living/Social environment, and Institutional contexts—to make practical actions to improve the adherence to the Mediterranean diet and lifestyle.

The concepts discussed in this article may be translated into *policy decisions* at the Institutional—Context level as follows (24):

1) Ensure that Food Systems are Sustainable along the entire food chain—from production to consumption; reduce food losses and waste. Involve multi-stakeholder partners. systemic policies designed to recognize food systems as complex adaptive systems (25).
2) Promote agriculture toward the best Sustainable Ecosystem services and practices. Reduce the use of pesticides and fertilizers (26).
3) Ensure the right of all members of the population to healthy, adequate, and affordable food.
4) Monitor regularly the safety of the food supply chain to be environmentally friendly and free of pathogens.
5) Legislate (and incentivize) the Food Industry to produce healthy (minimally processed foods), with less added sugars, trans fats, salt, and additives. *Informative Labeling* (27, 28). Ensure honest and transparent marketing with. No *junk food adverts to children*
6) Improve Public Health Education on healthy life styles, nutrition, cooking (Mediterranean Diet Patterns), and physical activity.

Once these policies are set in place, then implementation will follow by improving the living/social environment (relationships) and ensuring a healthy, safe external environment (institutional context) to affect the individual and, thereby, enhance adherence to the Mediterranean diet (29).

Finally, we note that diets should not be a list of do's and don'ts, but rather a pleasurable and tasty experience (Individual) in which we respect traditional and cultural preferences (Social Environment). We have to eat to live and not *vice versa*.

## Author contributions

LD and EB together conceived and developed the concept and contributed to the final manuscript. All authors contributed to the article and approved the submitted version.
